# Extracellular vesicles carrying miR-6836 derived from resistant tumor cells transfer cisplatin resistance of epithelial ovarian cancer via DLG2-YAP1 signaling pathway

**DOI:** 10.7150/ijbs.83264

**Published:** 2023-06-12

**Authors:** Yazhu Zou, Zitong Zhao, Jingjing Wang, Liying Ma, Yi Liu, Li Sun, Yongmei Song

**Affiliations:** 1 State Key Laboratory of Molecular Oncology, National Cancer Center/National Clinical Research Center for Cancer/Cancer Hospital, Chinese Academy of Medical Sciences and Peking Union Medical College, Beijing, China.; 2 Departments of Gynecological Oncology, National Cancer Center/National Clinical Research Center for Cancer/Cancer Hospital l & Shenzhen Hospital, Chinese Academy of Medical Sciences and Peking Union Medical College, Shenzhen, China.; 3 Departments of Gynecological Oncology, National Cancer Center/National Clinical Research Center for Cancer/Cancer Hospital, Chinese Academy of Medical Sciences and Peking Union Medical College, Beijing, China.; 4 State Key Laboratory of Molecular Oncology, Key Laboratory of Cancer and Microbiome, National Cancer Center/National Clinical Research Center for Cancer/Cancer Hospital, Chinese Academy of Medical Sciences and Peking Union Medical College, Beijing, China.

**Keywords:** Ovarian cancer, Cisplatin resistance, YAP1, DLG2, TEAD1, Extracellular vesicles, miR-6836

## Abstract

**Background:** Chemotherapy resistance is a significant cause for poor prognosis of epithelial ovarian cancer (EOC). However, the molecular mechanism of chemo-resistance remains unclear, and developing available therapies and effective biomarkers for resistant EOC is in urgent demand. Stemness of cancer cells directly results in chemo-resistance. Exosomal miRNAs rebuild tumor microenvironment (TME) and act as widely used clinical liquid biopsy markers.

**Methods:** In our study, high throughput screenings and comprehensive analysis were performed to screen for miRNAs, which were both up-regulated in resistant EOC tissues and related to stemness, and miR-6836 was identified accordingly.

**Results:** Clinically, high miR-6836 expression was closely correlated with poor chemotherapy response and survival for EOC patients. Functionally, miR-6836 promoted EOC cell cisplatin resistance by increasing stemness and suppressing apoptosis. Mechanistically, miR-6836 directly targeted DLG2 to enhance Yap1 nuclear translocation, and was regulated by TEAD1 forming the positive feedback loop: miR-6836-DLG2-Yap1-TEAD1. Furthermore, miR-6836 could be packaged into secreted exosomes in cisplatin-resistant EOC cells and exosomal miR-6836 was able to be delivered into cisplatin-sensitive EOC cells and reverse their cisplatin response.

**Conclusion:** Our study revealed the molecular mechanisms of chemotherapy resistance, and identified miR-6836 as the possible therapeutic target and effective biopsy marker for resistant EOC.

## Introduction

Ovarian cancer (OC) is the second most lethal cause of gynecologic cancer worldwide 1. Epithelial ovarian cancer (EOC) accounts for over 95% of OC. Moreover, due to its high incidence and mortality compared to other non-epithelial histological types, our research mainly focuses on EOC. Owing to lack of reliable early-stage screening methods, about 70% patients are diagnosed as stage III or IV 2-4. And upfront treatment mainly includes debulking surgery and platinum-based chemotherapy, while cisplatin is the first-line platinum-based drug for advanced EOC patients 5. Even though the initial response rate reaches 70%-75%, almost all patients will relapse and develop platium resistance eventually 6. Therefore, chemotherapy resistance is a crucial factor for recurrence and poor prognosis. EOC patients are divided into platinum-sensitive or platinum-resistant according to whether experiencing progressive disease with the cutoff at 6 months 7. Nevertheless, the underlying molecular mechanism remains to be explored, which is the theoretical basis for the development of therapeutic strategy and promising liquid biopsy markers for resistant EOC.

As the first FDA approved platinum-based anticancer drug 8, cisplatin functions by forming DNA-platinum adducts and cross-links, which lead to DNA damage, replication inhibition and cell cycle arrest in G2 phase 9. The cisplatin-resistance mechanism of EOC is quite complex, including efflux of cisplatin mediated by ABC transporters and other membrane transporters 10, increase of DNA damage repair 11, inhibition of apoptosis 12 and up-regulated stemness signaling 13. Stemness signaling has been reported to be closely related to chemo-resistance 14-15. Cancer stem cells (CSCs) is defined as a small population of cancer cells with the ability of regeneration, differentiation and resistance to chemotherapy or radiotherapy 16. Ovarian cancer stem cells (OCSCs) were first derived from the ascites of an EOC patient 17, and were further identified with markers including CD44, CD133, CD24, CD117 and ALDH1 18. Key transcription factors like Nanog, Oct4 and Sox2 and multiple stemness signaling containing Notch pathway, Wnt pathway, Hedgehog pathway and Hippo pathway have been proved to be of vital importance in the generation and maintenance of OCSCs 19.

MicroRNAs (miRNAs) are a class of single-stranded, small non-coding RNA molecules with the length about 21 nucleotides encoded by endogenous genes 20, and miRNAs can inhibit or degrade target mRNAs by competitively binding to their complementary target sequences 21. Considerable evidence has shown that the dysregulation of miRNAs is closely related to various diseases including cancer, and miRNAs have been reported to be tumor promotive or suppressive depending on their target mRNAs 22. Exosomal miRNAs can be transferred from donor cells to recipient cells via exosomes, which are defined as extracellular vesicles containing RNAs and proteins with diameters ranging from 30 to 150 nm 23. Based on the transferable feature of exosomes, exosomal miRNAs secreted by cancer cells or CSCs can interfere cells infiltrated in tumor microenvironment and promote chemo-resistance 24. In this work, we identified the underlying mechanism of an exsomal miRNA, miR-6836, and further explored the potential of miR-6836 as an effective diagnostic biomarker and therapeutic target for chemotherapy-resistant EOC patients.

## Results

### MiR-6836 is up-regulated in chemo-resistant EOC patients and is markedly associated with poor prognosis

**Figure 1:**
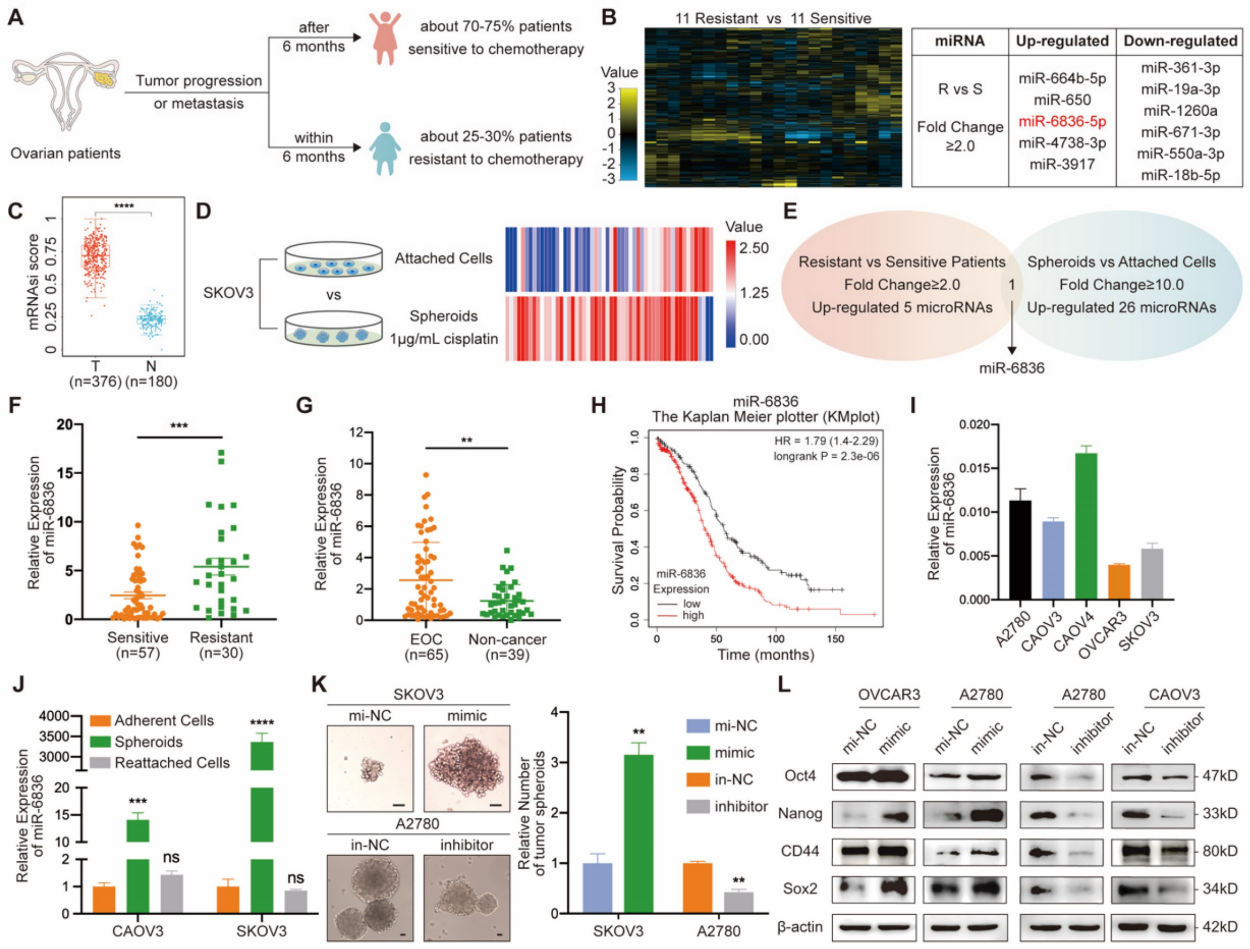
** miR-6836 is positively related to stemness signature in CSC and platinum resistance in EOC patients. a** Schematic diagram presenting the classification criteria of chemotherapeutic resistance. **b** Left: Heat map representing the expression levels of miRNAs in EOC tissues from 11 patients sensitive to chemotherapy and 11 resistant patients. Normalized expression levels are represented by color key; higher (yellow) or lower (blue). Right: Table listing miRNAs with significant changes (Fold change ≥ 2.0). **c** Stemness indices (mRNAsi score) of OV datasets and match TCGA normal data extracted by OCLR machine-learning algorithm derived from TCGA RNA-sequencing data. **d** Heat map of miRNA profile in SKOV3 attached cells and spheroids. Normalized expression levels are represented by colored bars; higher (red) or lower (blue). **e** Venn diagram of miRNAs up-regulated in chemotherapy resistant EOC patients compared with sensitive patients and SKOV3 spheroids than attached cells. **f** RT-qPCR analysis of miR-6836 expression in EOC tissues from chemotherapy sensitive patients (Sensitive, n=57) and resistant patients (Resistant, n=30). **g** RT-qPCR analysis of miR-6836 in serum samples from EOC patients (EOC, n=65) and healthy individuals or patients with benign ovarian tumor (Non-cancer, n=39). **h** Kaplan-Meier survival curves analysis of OS in EOC patients with different miR-6836 expression level from Kaplan Meier plotter (KMplot, http://kmplot.com/analysis). **i** RT-qPCR analysis of miR-6836 levels in EOC cells lines. **j** RT-qPCR analysis of miR-6836 levels in CAOV3/SKOV3 attached cells, spheroids and reattached cells. **k-l** A2780/OVCAR3/SKOV3 cells transfected with mimic-miR-6836 (mimic) or mimic-NC (mi-NC) and A2780/CAOV3 cells transfected with inhibitor-miR-6836 (inhibitor) or inhibitor-NC (in-NC). **k** Tumor spheroids formation assay to present the stemness of SKOV3 treated with mimic/mi-NC or A2780 cells with inhibitor/in-NC. Scale bar, 200 μm. The upper images show representative spheroids, and quantitative analyses are presented as histograms below. **l** Western blot analysis of Oct4, Nanog, CD44 and Sox2 expression in OVCAR3/A2780 treated with mimic/mi-NC or A2780/CAOV3 with inhibitor/in-NC. Results are presented as mean ± SEM; *P<0.05, **P<0.01, ***P<0.001.

In order to identify over-expressed miRNAs in EOC patients resistant to chemotherapy, twenty-two patients with EOC were enrolled, half of whom were sensitive to platinum-based chemotherapy and the other half with chemo-resistance (Fig. 1a). Five miRNAs up-regulated in EOC tissues from chemo-resistant patients were selected by miRNA array (Fig. 1b). Cancer progression is closely related to the acquisition of stemness features, which can be quantified by stemness indices (mRNAsi score) extracted by one-class logistic regression (OCLR) machine-learning algorithm from TCGA RNA-sequencing data 25. Stemness feature of OV were significantly higher than normal group (Fig. 1c). In order to screen for stemness related miRNA, SKOV3 spheroids were cultured and sequenced along with SKOV3 attached cells (Fig. 1d). Twenty-six miRNAs with higher expression in SKOV3 spheroids compared to attached cells were further screened out. By overlapping two groups of tumor-promoting miRNAs, miR-6836 was the specific miRNA that was both positively related to stemness and platinum-based chemo-resistance in EOC (Fig. 1e). To explore the clinical significance of miR-6836 in platinum-resistant patients, RT-qPCR was carried to quantify the expression of miR-6836 in 87 tumor tissues, which were derived from 57 resistant patients and 30 sensitive patients. MiR-6836 expression levels were observably higher in resistant EOC tissues than in sensitive EOC tissues (Fig. 1f). In order to further explore the potential of miR-6836 as a liquid biopsy biomarker, we analyzed miR-6836 serum expression levels in 65 EOC patients and 39 healthy individuals or patients with benign ovarian tumor using RT-qPCR, indicating that the serum expression levels of miR-6836 were significantly higher in EOC patients compared with healthy individuals and patients with benign ovarian tumor (Fig. 1g). Besides, based on KMplot online database, lower miR-6836 level was associated with longer overall survival (OS) in ovarian cancer patients (Fig. 1h). The expression of miR-6836 in EOC cell lines was shown in Figure 1i.

### Ectopic over-expression of miR-6836 enhances CSC properties

To investigate the correlation between miR-6836 expression and stemness phenotype of EOC cells, we cultured tumor spheroids with adherent EOC cells (CAOV3 and SKOV3) and reattached the OCSCs. RT-qPCR indicated that miR-6836 was remarkably over-expressed in EOC cells spheroids and dropped back to basal expression level after the reattachment of OCSCs (Fig. 1j), which revealed the close correlation between miR-6836 and EOC stemness. We transfected the EOC cells with miR-6836 mimic oligonucleotides (mimic) or negative control mimic miRNA (mi-NC) and with miR-6836 inhibitor oligonucleotides (inhibitor) or negative control inhibitor miRNA (in-NC). For the transfection efficiencies, see Supplementary Figure S1a-b. Tumor spheroids formation experiments showed that up-regulation of miR-6836 promoted the formation (number) and growth (volume) of EOC spheroids, and the inhibition of miR-6836 acted quite the contrary (Fig. 1k). Moreover, western blot quantified a series of protein markers for EOC cells stemness including Oct4, Nanog, CD44 and Sox2, and results showed that miR-6836 over-expression increased the expression of the mentioned stemness related markers and down-regulation of miR-6836 effected oppositely (Fig. 1l). Therefore, miR-6836 was proven with the ability of promoting the stemness of EOC cells.

### MiR-6836 upregulation facilitates cisplatin resistance of EOC cells *in vitro*

**Figure 2:**
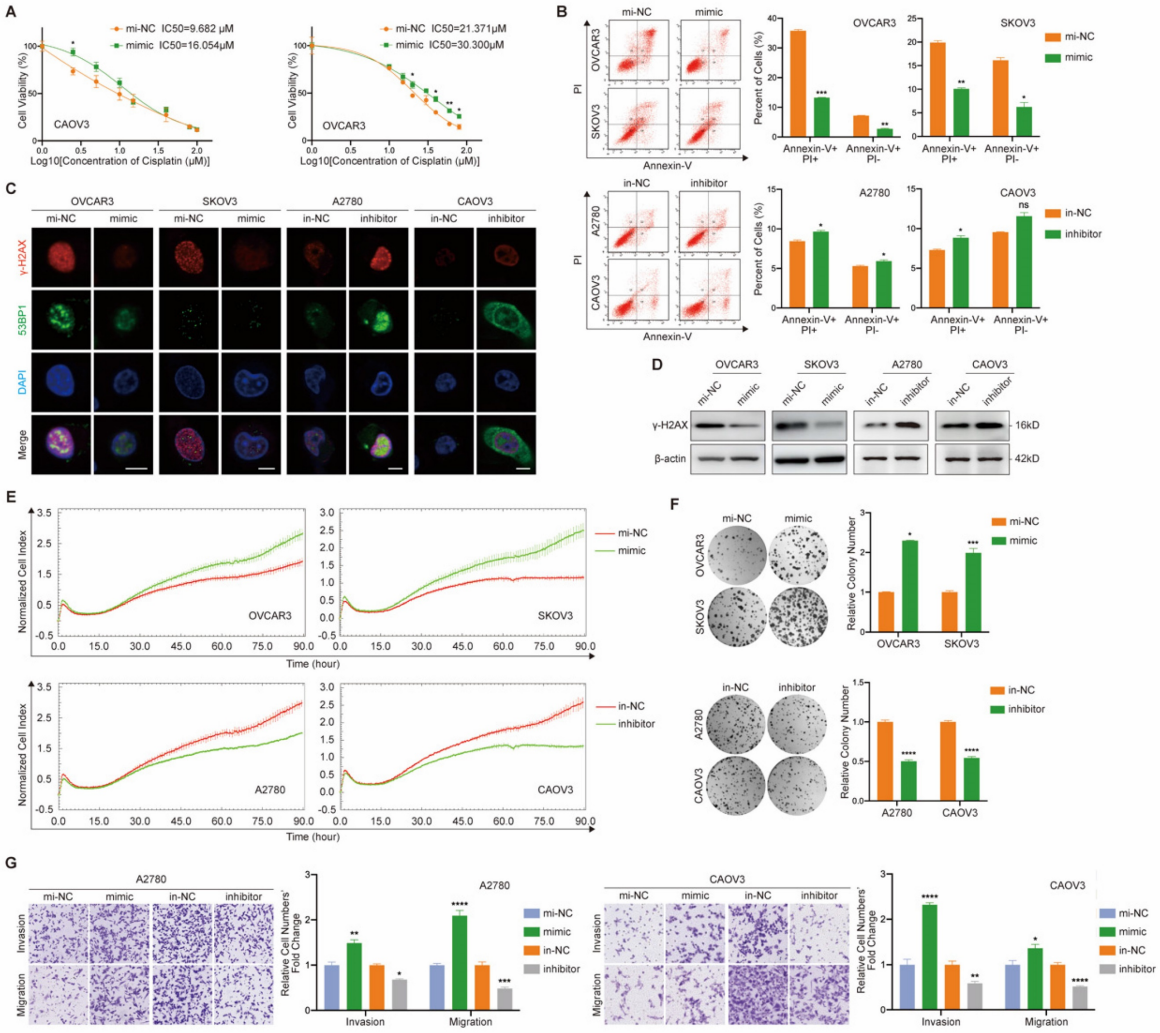
** miR-6836 induces cisplatin resistance of EOC cells. a** CCK8 assay to evaluate the cell viability of CAOV3/OVCAR3 transfected with mimic/mi-NC and further treated with cisplatin at various concentrations. IC50 calculation to evaluate the effect of mimic on the sensitivity of EOC cells to cisplatin by SPSS. **b** Flow cytometry assay to detect the influence of mimic or inhibitor on EOC cells apoptosis after treated with cisplatin for 24h. Representative images of apoptosis assay are shown on the left and quantitative data are presented as histograms. **c** Immunofluorescence assay for γ-H2AX and 53BP1 expression representing DNA damage caused by cisplatin treatment for 24h. Scale bar, 10 μm. **d** Western blot analysis of γ-H2AX expression in OVCAR3/SKOV3 treated with mimic/mi-NC or A2780/CAOV3 with inhibitor/in-NC. **e** Cell growth ability analysis of OVCAR3/SKOV3 transfected with mimic/mi-NC or A2780/CAOV3 with inhibitor/in-NC for 24h using the xCELLigence Real-Time Cell Analyzer (RTCA)-MP system. **f** Colony formation assay to assess the effect of mimic or inhibitor on cell growth and colony formation ability of EOC cells. Representative images of colony formation assay are shown on the left and quantitative analyses are presented as histograms on the right. **g** Transwell assay to analyze invasion and migration ability of A2780/CAOV3 transfected with mimic/mi-NC or inhibitor/in-NC. Representative images of transwell assay are shown on the left and quantitative analyses are presented as histograms. Results are presented as mean ± SEM; *P<0.05, **P<0.01, ***P<0.001.

With the purpose of exploring the cellular function of miR-6836, we examined the effect of miR-6836 on cisplatin resistance, cell proliferation, colony formation, invasion and migration of EOC cells. Due to the over-expression of miR-6836 in chemo-resistant EOC tissues and its stemness related characteristics, we explored the impact of miR-6836 on cisplatin resistance phenotype of EOC cells. Compared to control group cells, cells transfected with mimic acquired cisplatin resistance, which showed an increased IC50 (Fig. 2a) and decreased apoptosis induced by cisplatin (Fig. 2b). Besides, on account of the DNA damage effect caused by cisplatin, we detected the expression of indicators for DNA damage, γ-H2AX and 53BP1 by immunofluorescence (Fig. 2c) and quantified γ-H2AX by western blot (Fig. 2d), which revealed the DNA damage reduction of EOC cells over-expressing miR-6836. In the meantime, inhibitor could increase cisplatin sensitivity of EOC cells compared to in-NC. Cell proliferation, colony formation and transwell assays showed that EOC cells transfected with mimic exhibited more malignant phenotypes including the acceleration of cell proliferation, colony formation, invasion and migration compared with negative control, while inhibitor suppressed the phenotypes mentioned above (Fig. 2e-g). The findings above suggested the tumor-promoting role for miR-6836 in EOC cells.

### MiR-6836 promotes EOC stemness, tumor growth and peritoneal seeding *in vivo*

**Figure 3:**
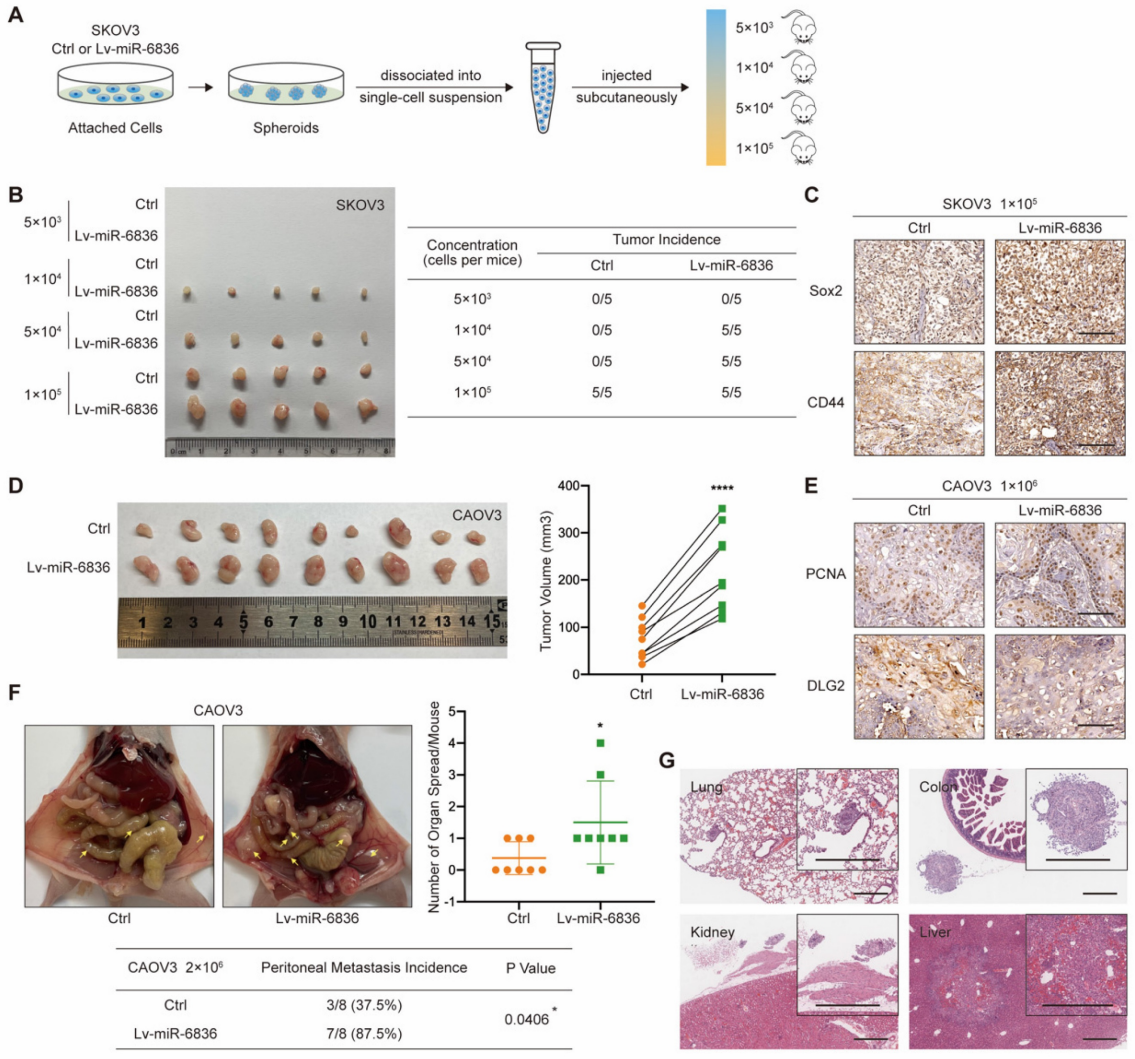
** miR-6836 induces tumor growth, metastasis and stemness of EOC cells *in vivo*. a-c** For spheroid cells tumorigenicity assay, four-week-old female NOD-SCID mice were injected with different concentration of single cell suspensions from SKOV3 Ctrl or Lv-miR-6836 spheroids. **a** Schematic diagram presents the experimental procedure of spheroid cells tumorigenicity assay. **b** The left image presents the tumor formation of SKOV3 ctrl or Lv-miR-6836 stem cells isolated from spheroids. The tumor incidence of concentration gradient is listed in the right table. **c** Representative IHC staining of Sox2 and CD44 in subcutaneous xenografts. Scale bar, 100 μm. **d,e** For subcutaneous tumor formation model, five-week-old female BALB/c nude mice were injected subcutaneously with 1×10^6^ CAOV3 cells infected with negative control lentivirus (Ctrl) or lenti-miR-6836 (Lv-miR-6836) in 100 μL per mouse (n=9). **d** Representative images of tumor tissues generated from Ctrl or Lv-miR-6836 cells are shown on the left and tumor volumes were measured and shown as scatter plot on the right. **e** Representative IHC staining of PCNA and DLG2 in subcutaneous xenografts. Scale bar, 100 μm. **f,g** For peritoneal metastatic model, five-week-old female BALB/c nude mice were injected intraperitoneally with 2×10^6^ Ctrl or Lv-miR-6836 CAOV3 cells in 100 μL per mouse (n=8). **f** Representative images of peritoneal metastatic tumors are exhibited on the left, the number of organ spread with metastatic tumors is counted and quantified using scatter plot (right) and peritoneal metastasis incidence is listed and analyzed in the table (below). **g** Hematoxylin-eosin (HE) staining of tumor metastatic organs is conducted and representative images of HE staining (including lung, colon, kidney and Liver) from Lv-miR-6836 group are shown. Scale bar, 500 μm. Results are presented as mean ± SEM; *P<0.05, **P<0.01, ***P<0.001.

With the purpose of assessing the role of miR-6836 in EOC *in vivo*, CAOV3 and SKOV3 cells were stably infected with negative control (Ctrl) or miR-6836 lentiviral vectors (Lv-miR-6836). EOC cells with Lv-miR-6836 infection had significantly higher miR-6836 expression than Ctrl cells (Supplementary Fig. S1c). To find out the effect of miR-6836 on stemness characteristic of EOC cells *in vivo*, we performed spheroid cells tumorigenicity assay. Ctrl and Lv-miR-6836 cells were cultured into stem cell spheroids. The spheroids were dissociated into single cells suspension, and were injected subcutaneously into 5 female NOD/SCID mice each group in four different concentration gradients (Fig. 3a). At the concentration of 5×10^3^ cells per mouse, neither group had tumor formation. At the concentration of 1×10^4^ and 5×10^4^ cells per mouse, all five mice in Lv-miR-6836 group had subcutaneous tumors, while none in Ctrl group. At the concentration of 1×10^5^ cells per mouse, all mice from both groups formed tumors, whereas tumors from Lv-miR-6836 group were observably larger than Ctrl group (Fig. 3b). The IHC results of 1×10^5^ group revealed that tumors developed from Lv-miR-6836 OCSCs had higher expression of EOC stemness markers including Sox2 and CD44 compared to Ctrl OCSCs (Fig. 3c). HE staining showed representative tumor tissues derived from spheroid cells tumorigenicity assay (Supplementary Fig. S2a).

To examine *in vivo* tumor growth, two groups of cells were injected subcutaneously into 9 female nude mice each so as to determine the effect of miR-6836 on cell proliferation *in vivo*. Tumor volumes in Lv-miR-6836 group were observably larger in contrast with Ctrl group (Fig. 3d). The results of IHC indicated that tumor generated from Lv-miR-6836 cells had higher PCNA and lower DLG2 expression (Fig. 3e). HE staining showed representative tumor tissues from subcutaneous transplantation assay (Supplementary Fig. S2b). Due to the widespread intraperitoneal metastases of EOC, Ctrl and Lv-miR-6836 cells were injected intraperitoneally into 8 nude mice each group. Lv-miR-6836 group exhibited significant higher metastatic potential in comparison with Ctrl group (Fig. 3f). The results of IHC indicated that liver and colon metastases generated from Lv-miR-6836 cells had higher Ki67 and lower DLG2 expression than Ctrl group (Supplementary Fig. S2c). HE staining showed representative metastases in different organs from Lv-miR-6836 group (Fig. 3g). According to mentioned results, the *invivo* malignant phenotype of miR-6836 included stemness, tumor growth and peritoneal seeding.

### DLG2 is the direct target of miR-6836 in EOC

**Figure 4:**
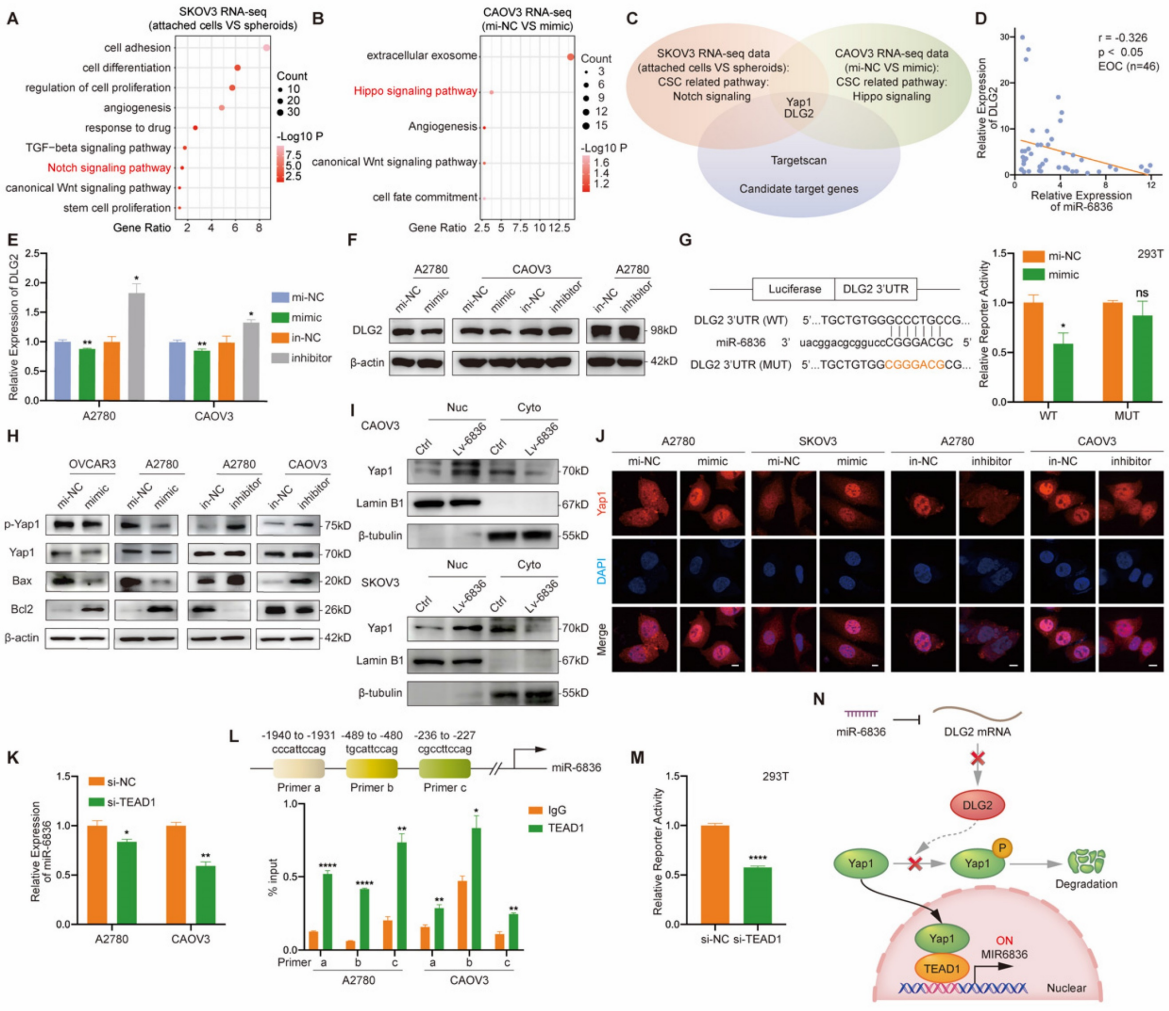
** miR-6836 regulates Yap1 subcellular localization by targeting DLG2. a** Bubble chart revealing the functional enrichment pattern of SKOV3 attached cells and spheroids. **b** Bubble chart revealing the functional enrichment pattern of CAOV3 transfected with mimic/mi-NC. **c** Venn diagram of putative miR-6836 candidate target genes predicted by Targetscan, and genes involved in Notch signaling and Hippo signaling pathway. **d** Spearman correlation analysis between DLG2 mRNA and miR-6836 expression levels in EOC tissues (n=46). The expression level of DLG2 and miR-6836 was measured by RT-qPCR. **e** RT-qPCR analysis of DLG2 expression level in A2780/CAOV3 treated with mimic/mi-NC or inhibitor/in-NC. **f** Western blot analysis of DLG2 expression in A2780/CAOV3 treated with mimic/mi-NC or inhibitor/in-NC. **g** The schematic diagram on the left shows the dual luciferase reporter plasmids construction containing wild type (WT) or mutated (MUT) miR-6836 binding site in DLG2 3' UTR. 293T cells were transfected with WT/MUT recombined luciferase report vector and mimic/mi-NC. Luciferase reporter activity was normalized to Renilla luciferase activity. **h** Western blot analysis of p-Yap1, Yap1, Bax, Bcl2 and β-actin expression in OVCAR3/A2780 treated with mimic/mi-NC or A2780/CAOV3 with inhibitor/in-NC. **i** Western blot analysis of Yap1 expression in the nucleus and cytoplasm of CAOV3/SKOV3 Ctrl or Lv-miR-6836. **j** Immunofluorescence assay for the subcellular localization of Yap1 caused by mimic/mi-NC or inhibitor/in-NC treatment. Scale bar, 10 μm. **k** RT-qPCR analysis of miR-6836 expression in A2780/CAOV3 treated with si-TEAD1/si-NC. **l** Schematic diagram shows the possible TEAD1 binding regions of miR-6836 promoter region. ChIP assay is performed using TEAD1 antibodies and IgG as the negative control. **m** Luciferase reporter assay: 293T cells were transfected with si-TEAD1/si-NC and recombined luciferase report vector containing miR-6836 promoter region. Luciferase reporter activity was normalized to Renilla luciferase activity. **n** The schematic diagram of miR-6836-DLG2-Yap1-TEAD1 positive feedback loops in regulating EOC cisplatin resistance. In resistant EOC cells, miR-6836 suppressed apoptosis and activated stemness by directly targeting DLG2, and further promoted Yap1 nuclear translocation and interaction with TEAD1. TEAD1 is both the transcriptional regulator and downstream for miR-6836. Results are presented as mean ± SEM; *P<0.05, **P<0.01, ***P<0.001.

In order to investigate how could miR-6836 promote cisplatin resistance in EOC, we first analysed the RNA sequencing data of SKOV3 attached cells and spheroids from Figure 1d, and Notch signaling pathway was found crucial in EOC cells spheroids formation (Fig. 4a). We further performed transcriptome sequencing on CAOV3 transfected with mimic/mi-NC, and enrichment analysis showed that Hippo signaling pathway was closely involved (Fig. 4b). Considering the enrichment results of the two mentioned transcriptome sequencing datasets, Yap1 was involved in both Notch and Hippo signaling pathway. Therefore, Yap1 involved signaling pathways are of vital importance in stemness feature formation and miR-6836 downstream. We further identified DLG2 as the putative target of miR-6836 by overlapping candidate genes predicted by computational tool Targetscan and genes involved in Yap1 related signaling pathways (Fig. 4c). MiR-6836 was demonstrated as a tumor promoter in EOC, so its target genes were supposed to be tumor suppressor genes. DLG2 was down-regulated in EOC compared to normal samples in match TCGA normal and GTEx database 26 (Supplementary Fig. S1d). DLG2, the selected putative target gene was proved to be negatively correlated to the expression level of miR-6836 in EOC patients (Fig. 4d and Supplementary Fig. S1e). Hence, we concentrated on DLG2 in the following research. In mimic treated EOC cells, increased miR-6836 decreased DLG2 in mRNA and protein level (Fig. 4e-f). Moreover, we constructed dual luciferase reporter plasmids containing wild type (WT) or mutated (MUT) miR-6836 binding site in DLG2 3' UTR. As shown in Figure 4g, miR-6836 suppressed the luciferase activity in WT plasmids but not in MUT plasmids. The experimental verification of regulatory relationships between miR-6836 and DLG2 and luciferase reporter assays indicated that miR-6836 directly targeted DLG2.

To further explore the molecular mechanism of miR-6836 by targeting DLG2, we examined the expression level of the reported downstream signaling of DLG2 by western blot based on the published mechanism of DLG2 as a tumor suppressor gene 27-28. We found that miR-6836 could promote Yap1 nuclear translocation by inhibiting phosphorylation of Yap1 without altering total Yap1 expression level, restrain apoptosis via up-regulating Bcl2 and down-regulating Bax, and advance stemness by positively regulating Oct4, Nanog, CD44 and Sox2 (Fig. 4h and Fig. 1l). Based on the mentioned western blot verification, we provided solid proof for Yap1 nuclear translocation mediated by miR-6836 via nuclear and cytoplasmic protein extraction (Fig. 4i) and immunofluorescence (Fig. 4j). Collectively, the results above proved that miR-6836 suppressed apoptosis and activated stemness by directly targeting DLG2, and then realized the function of inducing cisplatin resistance in EOC.

### TEAD1 acts as the transcriptional regulator of miR-6836 forming a TEAD1-miR-6836-DLG2-Yap1 positive feedback loop

In the following study, we intended to determine the upstream regulator for miR-6836. The findings above showed that miR-6836 stimulated the nuclear translocation of Yap1. Previous studies reported that hypo-phosphorylated Yap1 accumulated in the nucleus, bound to various transcription factors (TFs) and regulated target gene expression that controlled a range of biological events. The TEAD family was the most notable TFs that interacted with Yap1 29. Among the TEAD family, there was no significant difference in altered downstream of TEAD1-4 in EOC cells transfected with mimic. On the other hand, TEAD1 significantly affected overall survival (OS) of ovarian cancer patients based on TCGA database 26 (Supplementary Fig. S1g), so we further took TEAD1 as representative to verify its possibility as the putative miR-6836 upstream regulator. First and foremost, the regulatory relationship of TEAD1 to miR-6836 needed to be confirmed. RT-qPCR showed that TEAD1 down-regulation led to observably reduced expression of miR-6836 in EOC cells (Fig. 4k). And then, based on the putative binding sited in the miR-6836 promoter region predicted by JASPAR, ChIP assay was carried out and results confirmed the interaction between TEAD1 and miR-6836 promoter region (Fig. 4l). Finally, to explore the transcriptional activity of TEAD1 on miR-6836, a luciferase reporter plasmid containing the miR-6836 promoter region was constructed. Dual-luciferase reporter assay showed that the luciferase activity decreased markedly in TEAD1 down-regulated 293T cells (Fig. 4m). Based on the results above, TEAD1 is the transcriptional regulator for miR-6836 and therefore forms the positive feedback loop: miR-6836-DLG2-Yap1-TEAD1 (Fig. 4n).

### MiR-6836 confers aggressive EOC cell phenotypes through suppression of DLG2

**Figure 5:**
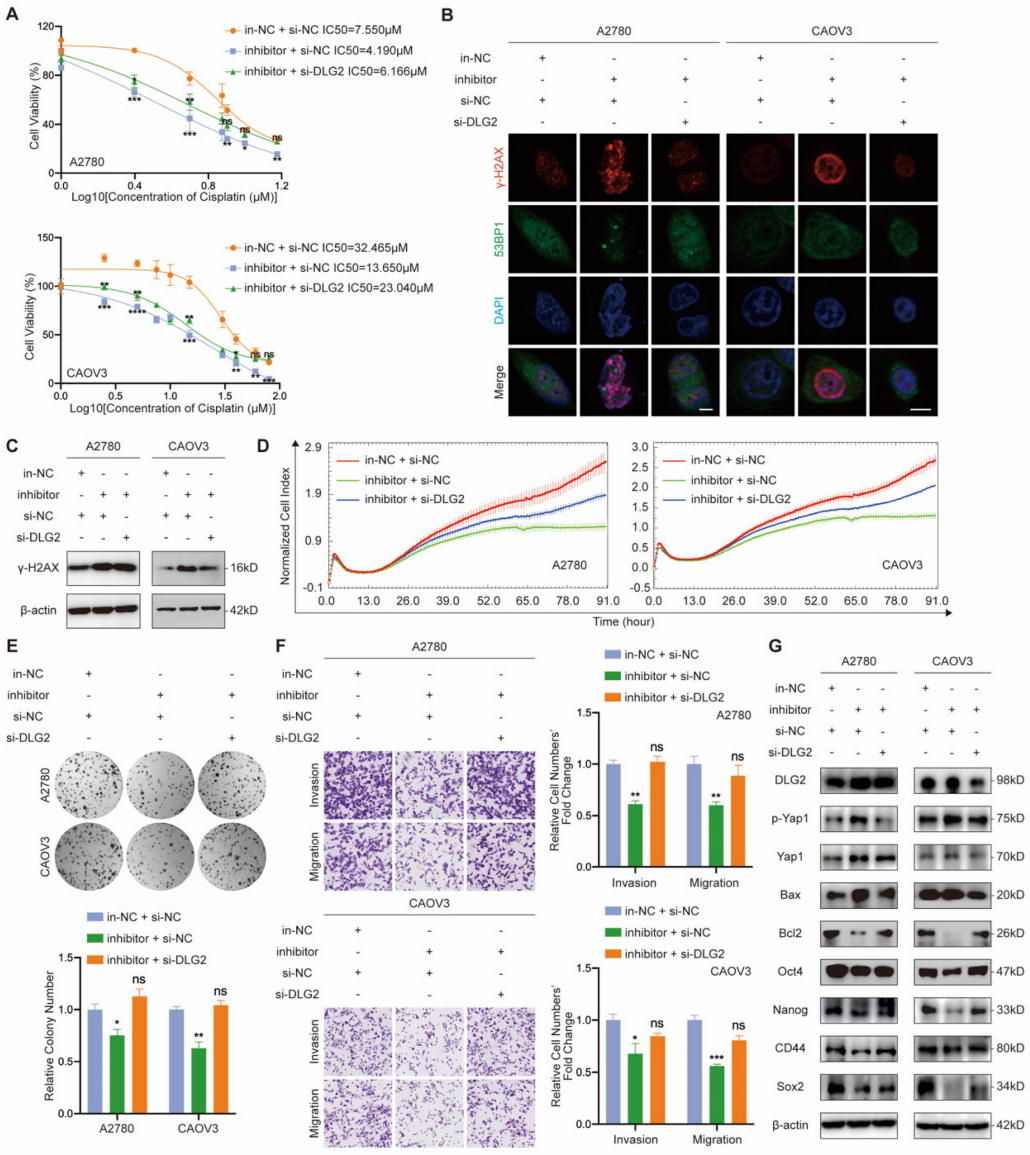
** miR-6836 increases cisplatin resistance by repressing DLG2. a** CCK8 assay to evaluate the cell viability of A2780/CAOV3 transfected with in-NC and si-NC, inhibitor and si-NC, or inhibitor and si-DLG2 and further treated with cisplatin at various concentrations. IC50 calculation to evaluate the effect of knocking down DLG2 on the basis of inhibitor on the sensitivity of EOC cells to cisplatin by SPSS. **b** Immunofluorescence assay for γ-H2AX and 53BP1 expression representing DNA damage caused by cisplatin treatment for 24h. Scale bar, 10 μm. **c** Western blot analysis of γ-H2AX expression in A2780/CAOV3 treated with inhibitor/in-NC and si-DLG2/si-NC. **d** Cell growth ability analysis of A2780/CAOV3 transfected with inhibitor/in-NC and si-DLG2/si-NC for 24h using the xCELLigence RTCA-MP system. **e** Colony formation assay to assess the effect of knocking down DLG2 on the basis of inhibitor on cell growth and colony formation ability of EOC cells. Representative images of colony formation assay are shown as above and quantitative analyses are presented as histograms as below. **f** Transwell assay to analyze invasion and migration ability of A2780/CAOV3 transfected with inhibitor/in-NC and si-DLG2/si-NC. Representative images of transwell assay are shown on the left and quantitative analyses are presented as histograms. **g** Western blot analysis of DLG2, p-Yap1, Yap1, Bax, Bcl2, Oct4, Nanog, CD44, Sox2 and β-actin expression in A2780/CAOV3 treated with inhibitor/in-NC and si-DLG2/si-NC. Results are presented as mean ± SEM; *P<0.05, **P<0.01, ***P<0.001.

In order to verify the regulatory relationship between miR-6836 and DLG2, we performed functional assays on EOC cells treated with in-NC or inhibitor, and si-NC or si-DLG2. Cisplatin resistance, cell proliferation, clonal formation and transwell phenotype assays indicated that inhibiting miR-6836 observably restrained the aggressive phenotype mentioned above, and knockdown DLG2 could rescue the suppression of the mentioned malignant phenotype mediated by miR-6836 inhibition (Fig. 5a-f). Similarly, down-regulation of DLG2 could reverse the reduced expression of oncogenetic downstream signaling and increased expression of tumor suppressive downstream signaling caused by miR-6836 down-regulation (Fig. 5g). The rescue findings confirmed that miR-6836 facilitated cisplatin resistance of EOC cells via directly targeting DLG2.

### Exosomal miR-6836 transfers chemo-resistance phenotype via converting DLG2 expression in recipient cell

**Figure 6:**
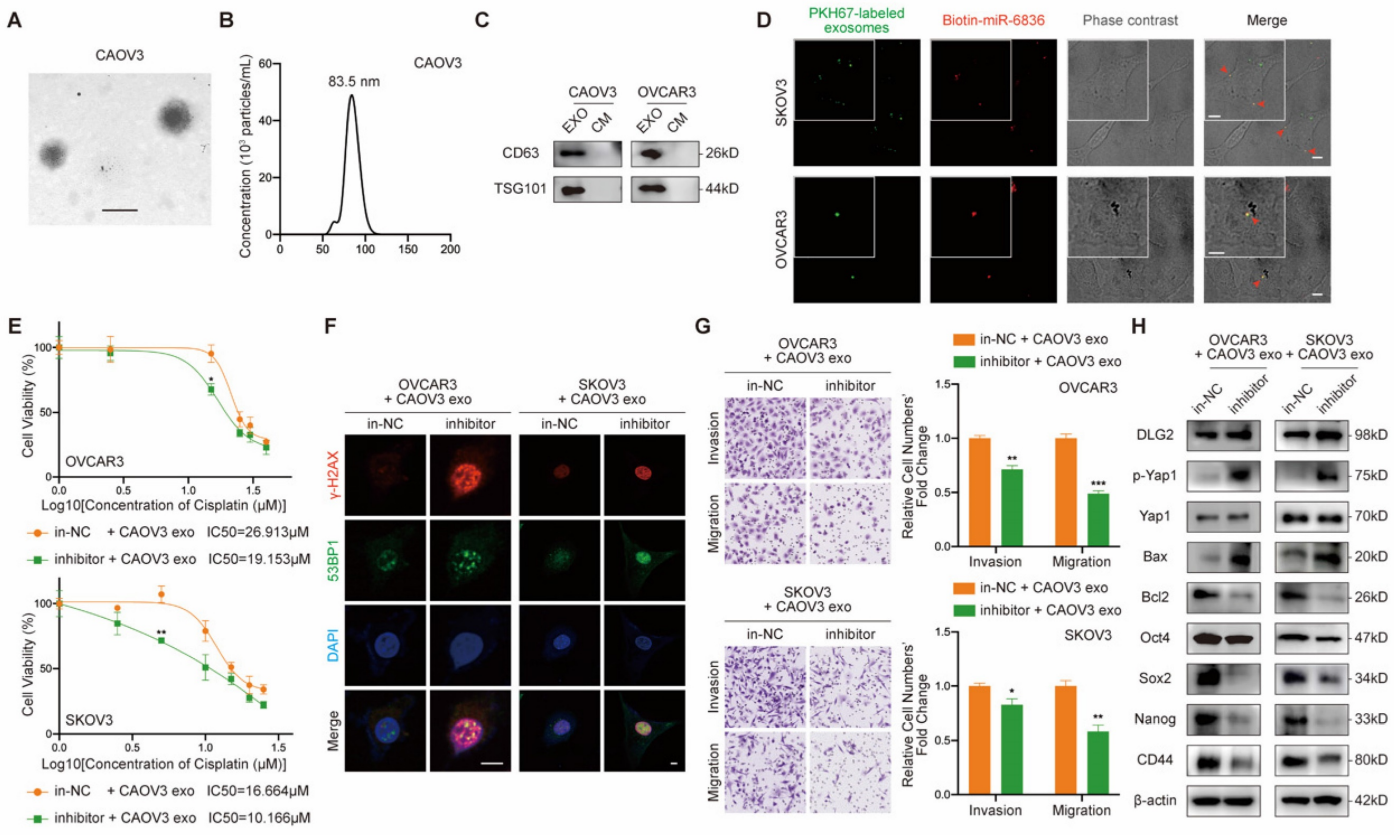
** Intercellular transfer of miR-6836 by exosomes disseminates cisplatin resistance. a** Representative CAOV3 exosome image under electron microscopy. Scale bar, 100 nm. **b** Nanoparticle tracking analysis measuring the diameter of CAOV3 exosomes. **c** Western blotting analysis of exosomal protein markers, CD63 and TSG101 expression in exosomes (EXO) and culture medium after exosome purification (CM) gathered from CAOV3 and OVCAR3 culturing. **d** Exosome internalization: CAOV3 exosomes (Green, PKH67-labeled) containing biotin-miR-6836 (Red, Streptavidin/RBITC-stained) were uptake by OVCAR3/SKOV3 (Phase contrast) cells. Scale bar, 10 μm. **e** CCK8 assay to evaluate the cell viability of OVCAR3/SKOV3 treated with inhibitor/in-NC and CAOV3 exosomes and further treated with cisplatin at various concentrations. IC50 calculation to evaluate the effect of exosomes on transferring the cisplatin sensitivity of EOC cells by SPSS. **f** Immunofluorescence assay for γ-H2AX and 53BP1 expression representing DNA damage caused by cisplatin treatment for 24h. Scale bar, 10 μm. **g** Transwell assay to analyze invasion and migration ability of OVCAR3/SKOV3 treated with inhibitor/in-NC and CAOV3 exosomes. Representative images of transwell assay are shown on the left and quantitative analyses are presented as histograms. **h** Western blot analysis of DLG2, p-Yap1, Yap1, Bax, Bcl2, Oct4, Nanog, CD44, Sox2 and β-actin expression in OVCAR3/SKOV3 treated with inhibitor/in-NC and CAOV3 exosomes. Results are presented as mean ± SEM; *P<0.05, **P<0.01, ***P<0.001.

Due to the known intercellular transferable feature of miRNA mediated by exosomes, we further explored the feasibility of transferring cisplatin resistance phenotype between EOC cells. Firstly, exosomes purified from EOC cells culture medium (CM) by ultracentrifugation were confirmed by its cup-shaped morphology characterized by transmission electron microscope (Fig. 6a), diameters ranging from 30-150nm analyzed by nanoparticle tracking analysis (Fig. 6b) and exosome markers CD63 and TSG101 identified by western blot (Fig. 6c). Next, we intended to figure out whether miR-6836 was able to be packaged into exosomes and transfered into recipient EOC cells. In donor EOC cells, transfected miR-6836 was labeled with biotin, and exosomes were isolated from CM, marked with PKH67, and incubated with recepient EOC cells. In recepient cells, biotin labeled miR-6836 fluoresced when treated with Streptavidin/RBITC, and the colocalization of miR-6836 and exosomes revealed that miR-6836 transferred from higher to lower expressed EOC cells mediated by exosomes (Fig. 6d).

Then, we further explored whether the cisplatin resistance phenotype could be transfered to recipient sensitive EOC cells by exsomoal miR-6836. In order not to affect exosome inclusions, EOC cells with relatively low miR-6836 expression were transfected with in-NC or inhibitor and treated with exosomes purified from CM of CAOV3, an EOC cell line with high constitutive miR-6836 expression. As expected, inhibitor could not lead to significant difference in miR-6836 expression without CAOV3 exosomes treatment, and yet the miR-6836 expression was notably different when treated with both inhibitor and exosomes (Supplementary Fig. S1f). CCK8 assay, immunofluorescence and transwell assay verified the exosome-mediated delivery of malignant phenotypes including cisplatin resistance, invasion and migration from resistant EOC cells to sensitive cells (Fig. 6e-g). Western blot proved that exosomal miR-6836 induced cisplatin resistance in sensitive cells via altering the expression of its target gene DLG2 and a series of downstream signaling pathways including apoptosis and stemness (Fig. 6h). Thus, these findings revealed that exosomal miR-6836 transfered cisplatin resistance through altering DLG2 expression in recepient EOC cells.

### The combined treatment of miR-6836 antagonist and cisplatin alleviates cisplatin resistance *in vivo*

**Figure 7:**
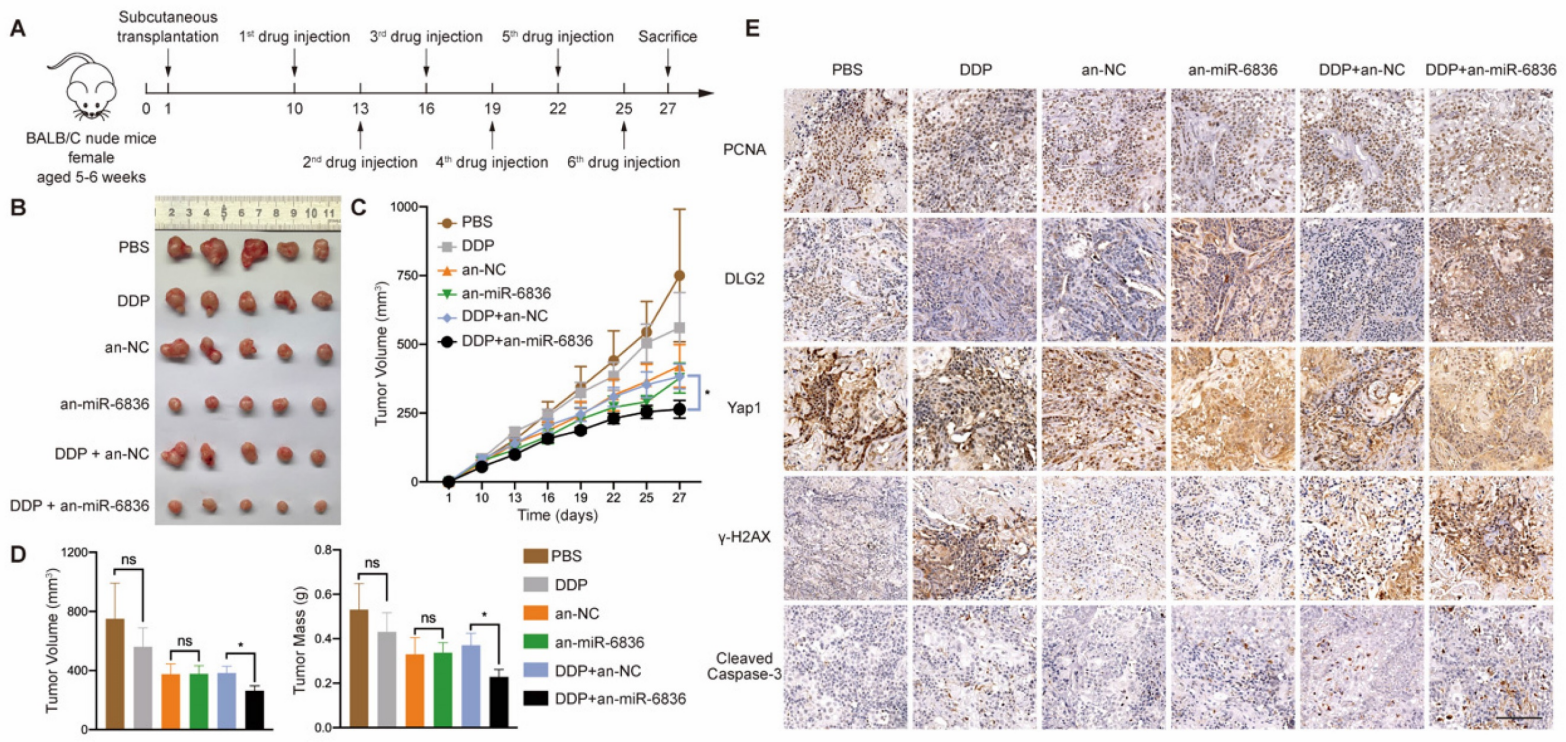
** The combinational treatment of antagomir-miR-6836 and cisplatin enhances cisplatin sensitivity for EOC *in vivo*. a** Schematic model presenting the drug combination animal model. **b** Representative tumor images after treatments including PBS, DDP, antagomir-NC, antagomir-miR-6836, and the combination of DDP and antagomir-NC or antagomir-miR-6836 (n=5). **c** Growth curves of tumors after treatments (n=10). **d** Tumor volume and mass analysis (n=10). **e** Representative IHC staining of PCNA, DLG2, Yap1, γ-H2AX and cleaved caspase-3 in CAOV3 xenografts with treatments. Scale bar, 100 μm. Results are presented as mean ± SEM; *P<0.05, **P<0.01, ***P<0.001.

In order to assess the therapeutic potential of miR-6836 antagonist in reversing cisplatin resistance, EOC subcutaneous transplantation mouse model was established. Antagomir-miR-6836 (an-miR-6836) is a chemically modified miR-6836 antagonist, which inhibits miR-6836 from complementary paired with DLG2 3'UTR by competitively binding to mature miR-6836 *in vivo* (Fig. 7a). After drug injection, the combination treatment of cisplatin and an-miR-6836 had maximized effect compared to cisplatin treatment alone or the combination of cisplatin and antagomir-NC (an-NC) (Fig. 7b-d). IHC results proved that an-miR-6838 treated tumors had higher DLG2 expression, and lower nuclear localized Yap1 level. In addition, tumors treated with both cisplatin and an-miR-6836 had lower PCNA level representing lower proliferation and higher γ-H2AX and cleaved caspase-3 expression characterizing higher apoptosis level than cisplatin or an-NC group (Fig. 7e). Therefore, these results confirmed the potential for miR-6836 antagonist to reverse cisplatin resistance *in vivo*.

## Discussion

As the major cause for tumor recurrence and metastasis, chemo-resistance remains to be an unsolved problem for EOC patients. Therefore, to elucidate underlying mechanisms, identify promising biomarkers and finding out possible solutions are urgently demanded in clinical diagnosis and treatment. On account of offering a viable strategy for the current clinical dilemma of EOC, our research identified a tumor progressive miRNA, miR-6836. MiR-6836 is up-regulated in chemo-resistant EOC and functionally contributes to cisplatin-resistant phenotype. MiR-6836 realizes its function via inhibiting the expression of its target gene, DLG2 and further increasing the dephosphorylation of Yap1 and its accumulation in the nucleus. TEAD1 acts as both downstream and transcriptional upstream of miR-6836 via interacting and co-activating with Yap1. In the meantime, it also accelerates the synthesis of miR-6836, and therefore forms the positive feedback loop of miR-6836-DLG2-Yap1-TEAD1. We also put forward a new strategy of enhancing the sensitivity of platinum-based chemotherapy in EOC using antagomir-miR-6836. In *in vivo* drug combination studies, the combination treatment of antagomir-miR-6836 and cisplatin suppresses tumor growth remarkably indicating the potential of antagomir-miR-6836 as the possible strategy for improving chemosensitivity.

Our study was the first to report the function and mechanism of miR-6836 as an oncogenic gene and demonstrated that resistant EOC cells released EVs-riched miR-6836 to promote stemness and cisplatin resistance of sensitive EOC cells *in vivo* and *in vitro*. MiR-6836 was first reported as a tumor suppressor in head and neck cancer related to stemness 30. A recent study proved that miR-6836 was down regulated in exosomes of both osimertinib resistant plasma and M2 type tumor-associated macrophage (TAM) in non-small cell lung carcinoma and functioned via MSTRG.292666.16/miR-6386/MAPK8IP3 axis 31. However, different from previous findings, our research identified miR-6836 as a stemness related tumor-promoting factor in EOC. According to our findings, miR-6836 was significantly up-regulated in EOC tumor tissues and sera compared to healthy individuals. Besides, miR-6836 had higher expression level in chemo-resistant EOC tissues and EOC spheroids than sensitive patients and EOC attached cells. Hence, we put forward the vital significance of miR-6836 in chemo-resistance EOC.

Along with the recognition and development of tumor microenvironment (TME), TME has been extensively proven as an interactive ecosystem that promotes tumor growth and metastasis in EOC 32 and various other tumor types 33-35. The intercellular communication between cancer cells and other TME components include contact-dependent and contact-independent approaches, which include extracellular vesicles (EVs) and soluble molecules such as cytokines 36. Therein, EVs have been reported to promote the formation of pre-metastatic niche 37, chemo-resistant 38 and immune evasion 39 TME in EOC. The communication between EOC cells and macrophages in EOC resistance has been reported 40-42. Our study first reported the function and mechanism of the transmission of stemness and chemo-resistance phenotypes in TME via microRNA carried by EVs in TME between EOC cells. We explored and verified that miR-6836 could be packed into extracellular vesicles (EVs) in EOC cells, secreted in TME and transferred chemo-resistance phenotype via converting DLG2 expression in recipient sensitive EOC cell. In addition to the domestication of sensitive EOC cells that has been verified in the research, the tumor promoting induction of various TME component cells via exosomal microRNAs remains to be further verified and explored.

Revealing the underlying molecular mechanisms of chemotherapy resistance is of vital importance for developing ideal biomarkers and solutions for EOC chemo-resistance. We screened out the target gene of miR-6836, DLG2 and further explored the dominant axis. Previous studies suggested that as a tumor suppressor gene, DLG2 functioned by inhibiting stemness and promoting apoptosis in OC 27, and DLG2 suppressed colorectal cancer via enhancing phosphorylation of Yap1 in colorectal cancer 28. In accordance with mentioned previous results, we further proved that as the target gene of miR-6836, DLG2 restrained the nuclear translocation of Yap1 in EOC. Yap1 was reported as an oncogene and was related to poor prognosis of EOC patients 43. When Hippo-YAP pathway was dysregulated, Yap1 translocated into nucleus and activated stemness signaling by interacting with TEAD1 29. According to our results, the expression of miR-6836 was up-regulated with the cooperation of Yap1/TEAD1 complex, which formed a positive feedback axis. Over-expression of exosomal miR-6836 could contribute to the intercellular transmission of cisplatin-resistance between EOC cells via reducing the expression of DLG2, promoting the nuclear translocation of Yap1/TEAD1 and further leading to miR-6836 over-expression in cisplatin sensitive recipient EOC cells.

Cisplatin resistance is one of the most urgent problems in EOC clinical treatment. About 25-30% EOC patients have to suffer from the side effects of platinum-based drugs without benefiting from them 6. In consequence, identifying prospective biomarkers for distinguishing chemo-resistance and providing possible therapeutic measures are of vital importance. MiRNAs combine the features of both stable existence and exosomal secretion, which makes miRNAs a reliable type of biomarkers. Based on our results, miR-6836 has the potential of becoming the liquid biopsy marker for EOC diagnosis and the biopsy biomarker for EOC cisplatin resistance detection. Besides, antagomir miR-6836 can help reversing EOC cisplatin resistance, which presents its clinical benefits for resistant EOC patients.

Even though we are the first to uncover the contribution of miR-6836 in EOC resistance, there are several limitations that need to be discussed. First, due to the long term required for determining the chemotherapy sensitivity for EOC patients, the number of EOC tissue biopsy and serum specimens is limited. Expanding the sample capacity of EOC patients presents the possibility of miR-6836 as the liquid biopsy markers for EOC chemotherapy sensibility, and further increases the credibility of our conclusions. Besides, herein the *invivo* and *invitro* functional experiments and molecular mechanism exploration are based on cisplatin. When it comes to EOC patients' recruitment criteria, the choice of postoperative chemotherapy regimens is not strictly limited to cisplatin but includes other platinum-based drugs like nedaplatin and carboplatin. Thirdly, miRNAs form a complex interacting network, and a single miRNA involves several downstream target pathways. In this study, we screen out the key miRNA, miR-6836 on account of two filters: stemness characteristic and up-regulation in resistant EOC tissues, and first present the miR-6836/DLG2 axis. However, an effective miRNA panel can realize better prediction of chemotherapy sensitivity compared to a single miRNA. In conclusion, we demonstrate that exosomal miR-6836 can function as both biomarkers for EOC chemo-resistance and therapeutic targets to overcome cisplatin resistance.

## Methods

### Cell culture

All cell lines including A2780, CAOV3, CAOV4, OVCAR3, SKOV3, 293T cells were purchased from China Infrastructure of Cell Line Resource (Beijing, China). A2780 and OVCAR3 cells were cultured in Roswell Park Memorial Institute-1640 (RPMI-1640) supplemented with 10% fetal bovine serum (FBS) and antibiotics. CAOV3, SKOV3, 293T were cultured in Dulbecco's modified Eagle's medium (DMEM) supplemented with 10% FBS and antibiotics. CAOV-4 were cultured in DMEM supplemented with 20% FBS and antibiotics. All cell lines were maintained at 37 °C in a 5% CO_2_ incubator.

### OC patients, OC tissue and blood serum samples

A total of 87 samples of human OC tissues, 65 samples of human OC blood serums and 39 samples of healthy individuals' and benign ovarian tumor patients' blood serums were collected at at the Departments of Gynecological Oncology, National Cancer Center/National Clinical Research Center for Cancer/Cancer Hospital l & Shenzhen Hospital, Chinese Academy of Medical Sciences and Peking Union Medical College after obtaining the subjects' informed consent from October 2017 to November 2020. For chemotherapeutic response, 30 (34.48%) patients were resistant to platinum-based chemotherapy and 57 (65.52%) were sensitive to the same treatment. PFS was calculated at the time from the date of surgery to the occurrence of progression, or relapse. OS was measured as the length of time from the initiation of surgery to death from any cause or until the most recent follow-up. PFS less than 6 months was defined as resistant to the last chemotherapy, and more than 6 months was defined as sensitivity to the last chemotherapy.

### MicroRNA Array and mRNA Array

The Agilent/Affymetrix microarray was used for miRNA/mRNA expression profiles (CapitalBio, Beijing, China).

### RNA extraction, RT-PCR and quantitative real-time PCR

Total RNA was isolated from frozen fresh tissue or EOC cell lines by using NCMzol Reagent and RNAExpress Total RNA Kit (NCM Biotech, Suzhou, China). Serum miRNA was purified by adding 1mL NCMzol Reagent, 100uL serum and 1ng external control (Mus musculus lncRNA Gm13008-201 synthesized by *in vitro* transcription). RT-PCR and qPCR were conducted using EasyScript All-in-One First-Strand cDNA Synthesis SuperMix for qPCR (One-Step gDNA Removal) and PerfectStart Green qPCR SuperMix purchased from TransGen Biotech (Beijing, China). Relative expression level was normalized to U6 levels for cellular miR-6836, external control (lncRNA Gm13008-201) for exosomal miR-6836 and GAPDH for cellular DLG2 mRNA. Bulge-Loop miR-6836 qRT-PCR Primer Set was purchased from RiboBio (Guangzhou, China) and other qPCR primers used are listed in Table S1.

### Tumor Spheroids Formation Assays

EOC cells were seeded in ultra-low attachment 6 well plates (Corning, Kennebunk ME, USA) and cultured in serum free DMEM/F12 supplemented with 2% B27 Supplement (50×) (Gibco, Grand Island NY, USA), basic fibroblast growth factor (bFGF, Sigma-Aldrich, Saint Louis MO, USA) and epidermal growth factor (EGF, Sigma-Aldrich) with the final concentration of 20ng/μL at the temperature of 37 °C in a 5% CO_2_ incubator. Spheroids were recorded and counted under microscope after 2-4 weeks.

### Oligonucleotide transfection

MiR-6836 mimic or inhibitor and siRNA for DLG2, and TEAD1 were purchased from RiboBio (Guangzhou, China). Cell transfection was performed using Lipofectamine 2000 reagent (Invitrogen, Carlsbad CA, USA).

### Western blotting

Western blot was performed as previously described 44. The antibodies included anti-Oct4 (Bioss, Beijing, China, bs-0830R, 1:1000), anti-Nanog (Proteintech, Wuhan, China, 14295-1-AP, 1:1000), anti-CD44 (Proteintech, 15675-1-AP, 1:1000), anti-Sox2 (Proteintech, 11064-1-AP, 1:1000), anti-γ-H2AX (Abcam, Cambridge, UK, ab26350, 1:1000), anti-DLG2 (Affinity Biosciences, Suzhou, China, DF3995, 1:1000), anti-p-Yap1 (CST, Boston MA, USA, 4911S, 1:1000), anti-Yap1 (Proteintech, 13584-1-AP, 1:1000), anti-Bax (CST, B8429, 1:1000), anti-Bcl2 (Proteintech, 60178-1-Ig, 1:1000), anti-β-tubulin (Sigma-Aldrich, T5201, 1:1000), anti-Lamin B1 (Santa, Dallas TX, USA, sc-365962, 1:1000), anti-TEAD1 (ABclonal, Wuhan, China, A13366, 1:1000), anti-β-actin (Santa, sc-8432, 1:4000), CD63 (Proteintech, 25682-1-AP, 1:1000), and TSG101 (Proteintech, 28283-1-AP, 1:1000).

### Transwell migration/invasion assays

Cell migration and invasion assays were performed as previously described 44.

### Colony formation assay

Colony formation assay was conducted as previously described 44.

### Drug sensitivity detected by CCK8 assay

After 5×10^3^ cells were seeded in 96-well plates for 12h, cells were treated with cisplatin at different concentration gradients for 24h. Then cell viability was determined by Cell Counting Kit-8 (NCM Biotech, Suzhou, China). The growth-inhibitory curves were charted by CCK8, and the half maximal inhibitory concentration (IC50) representing the cisplatin concentration when cell viability is 50% was calculated.

### Apoptosis detected by flow cytometry assay

Cells were stained with AnnexinV and PI using Annexin V-FITC/PI Apoptosis Detection Kit (KeyGEN BioTECH, Nanjing, China), and then apoptosis ratio was analyzed using multicolor flow cytometer, LSRII (BD Biosciences, Franklin Lakes NJ, USA).

### Immunofluorescence assay

For immunofluorescence assay, primary antibodies included anti-γ-H2AX (Abcam, ab26350, 1:200), anti-53BP1 (Abcam, ab36823, 1:100), and Yap1 (Proteintech, 13584-1-AP, 1:50) and fluorescent secondary antibody included Alexa Flour 594 anti-mouse secondary antibody, Alexa Flour 488 anti-rabbit secondary antibody and Alexa Flour 594 anti-rabbit secondary antibody (ZSGB-BIO, Beijing, China). Immunofluorescence assay was conducted according to the manufacturer's protocol (Abcam, Immunocytochemistry and immunofluorescence protocol). Images were collected using laser‐scanning confocal microscope.

### Retroviral infection

The lentivirus for miR-6836 was purchased from GeneChem (Shanghai, China) and cells were infected based on the manufacturer's instructions.

### Animal experiments

For spheroid cells tumorigenicity assay, four-week-old female NOD-SCID mice were purchased from Weitonglihua (Beijing, China), and experimental procedures were recorded in Result 3.

For subcutaneous or peritoneal metastatic model, five-week-old female BALB/c nude mice were purchased from Beijing HFK Bioscience (Beijing, China). Ctrl and Lv-miR-6836 CAOV3 cells were injected subcutaneously (1×10^6^ cells per mouse) or intraperitoneally (2×10^6^ cells per mouse) into nude mice. Mice were sacrificed after 30 days and the volume of subcutaneous tumor tissues were measured. Tumor tissues and abdominal organs from intraperitoneal metastasis model were fixed in formaldehyde solution, embedded in paraffin and sectioned into slices for histological examination.

For drug combination animal model, antagomir-miR-6836 were purchased from RiboBio (Guangzhou, China). CAOV3 (2×10^6^ cells per mouse) were injected subcutaneously into the left axilla of nude mice. After 10 days, the nude mice formed tumors with the approximate volume of 75 mm^3^. PBS or cisplatin were injected intraperitoneally and antagomir-NC or antagomir-miR-6836 were dosed through tail vein.

### Plasmid construction

The DLG2 3'UTR (WT) or mutant (MUT) with a predicted miR-6836 target sequence was inserted downstream of the firefly luciferase gene in the pmirGLO vector.

The miR-6836 promoter region (-2000 bp ~ 0 bp) was inserted upstream of the firefly luciferase gene in the pmirGLO vector.

### Luciferase reporter assay

The Luciferase reporter assay was performed with the Dual-Luciferase Reporter Assay System (Promega, Madison WI, USA) as previously described 44.

### Nuclear and cytoplasmic protein extraction assay

Nuclear and cytoplasmic protein extraction assay was conducted using Nuclear and Cytoplasmic Protein Extraction Kit (Beyotime, Shanghai, China).

### Chromatin immunoprecipitation assays (ChIP) assay

ChIP assays were performed using a PierceTM Magnetic ChIP Kit (Thermo ScientificTM, Shanghai, China). Immunoprecipitation was performed with anti-TEAD1 antibodies (ABclonal, A13366). Primers (Primer a, b, c) involved in qRT-PCR were listed in Table S1.

### Exosome purification, identification and internalization

Exosomes were isolated with ultracentrifugation method. Cells were cultured in exosome-free culture medium. The supernatant was collected after incubation for 48 h, centrifuged at 1 000 g for 10 min, 12 000 g at 4°C for 30 min and then ultracentrifuged at 4°C at 110 000 g for 90 min. Exosomes were identified by observation under transmission electron microscope (TEM‐1400 Plus) and nanoparticle tracking analysis using NanoSight NS300 instrument (Malvern Instruments Ltd, UK).

In order to examine exosome internalization, biotin-miR-6836 was synthesized in Shanghai Generay Biotech (Shanghai, China). Donor cells were transfected with biotin-miR6836 and exosomes were collected from supernatant of donor cells 48h after transfection. Then exosomes were labeled with PKH67 (Sigma-Aldrich) according to manufacturer's protocols, and incubated with recepient cells for 12h. Recipient cells were fixed and stained with Streptavidin/RBITC (Bioss, bs-0437P-RBITC). Images were collected using laser‐scanning confocal microscope.

### Statistical analysis

The data were presented as means ± SEM and the differences between groups were analyzed using unpaired two-tailed Student's t test using the GraphPad Prism 8. Spearman correlation was used to assess the correlation between two involved variables. The statistical analyses were carried out using GraphPad Prism 8 and SPSS 23.0 software. For the significance of difference, p < 0.05 represented a significant difference. ns, not significant; *, P < 0.05; **, P < 0.01; ***, P < 0.001.

## Supplementary Material

Supplementary figures and table.

## Abbreviations

EOC: Epithelial ovarian cancer
OC: Ovarian cancer
TME: Tumor microenvironment
FDA: Food and drug administration
CSCs: Cancer stem cells
OCSCs: Ovarian cancer stem cells
miRNAs: MicroRNAs
mRNAsi: Stemness indices obtained from transcriptomic features
OCLR: One-class logistic regression
KMplot: Kaplan Meier plotter
OS: Overall survival
PFS: Progression free survival
WT/MUT: Wild type or mutated miR-6836 binding site in DLG2 3' UTR
TFs: Transcription factors
CM: EOC cells culture medium
EVs: Extracellular vesicles
TAM: Tumor-associated macrophage
mimic: MiR-6836 mimic oligonucleotides
mi-NC: Negative control mimic miRNA
inhibitor: MiR-6836 inhibitor oligonucleotides
in-NC: Negative control inhibitor miRNA
Ctrl: Negative control lentiviral vectors
Lv-miR-6836: MiR-6836 lentiviral vectors
si-NC: Negative control siRNA
si-DLG2: DLG2 siRNA
si-TEAD1: TEAD1 siRNA
an-miR-6836: Antagomir-miR-6836
an-NC: Antagomir-NC
DLG2: Discs Large MAGUK Scaffold Protein 2
Yap1: Yes1 Associated Transcriptional Regulator
p-Yap1: Phospho-YAP1
TEAD1: TEA Domain Transcription Factor 1
Oct4: POU Class 5 Homeobox 1
Sox2: SRY-Box Transcription Factor 2
Bcl2: BCL2 Apoptosis Regulator
Bax: BCL2 Associated X, Apoptosis Regulator
ABC transporters: ATP Binding Cassette Subfamily transporters
RPMI-1640: Roswell Park Memorial Institute-1640
FBS: Fetal bovine serum
DMEM: Dulbecco's modified Eagle's medium
CCK8: Cell Counting Kit-8
IC50: The half maximal inhibitory concentration
ChIP: Chromatin immunoprecipitation
