# Supplementary material for: Extracellular vesicles carrying miR-6836 derived from resistant tumor cells transfer cisplatin resistance of epithelial ovarian cancer via DLG2-YAP1 signaling pathway
Source: Int J Biol Sci. 2023 Jun 12;19(10):3099–114. doi: 10.7150/ijbs.83264 (PMC10321283; doi:10.7150/ijbs.83264)
Supplement: Supplementary file 1 — Supplementary figures and table. [file ijbsv19p3099s1.pdf]

Supplementary Figure 1

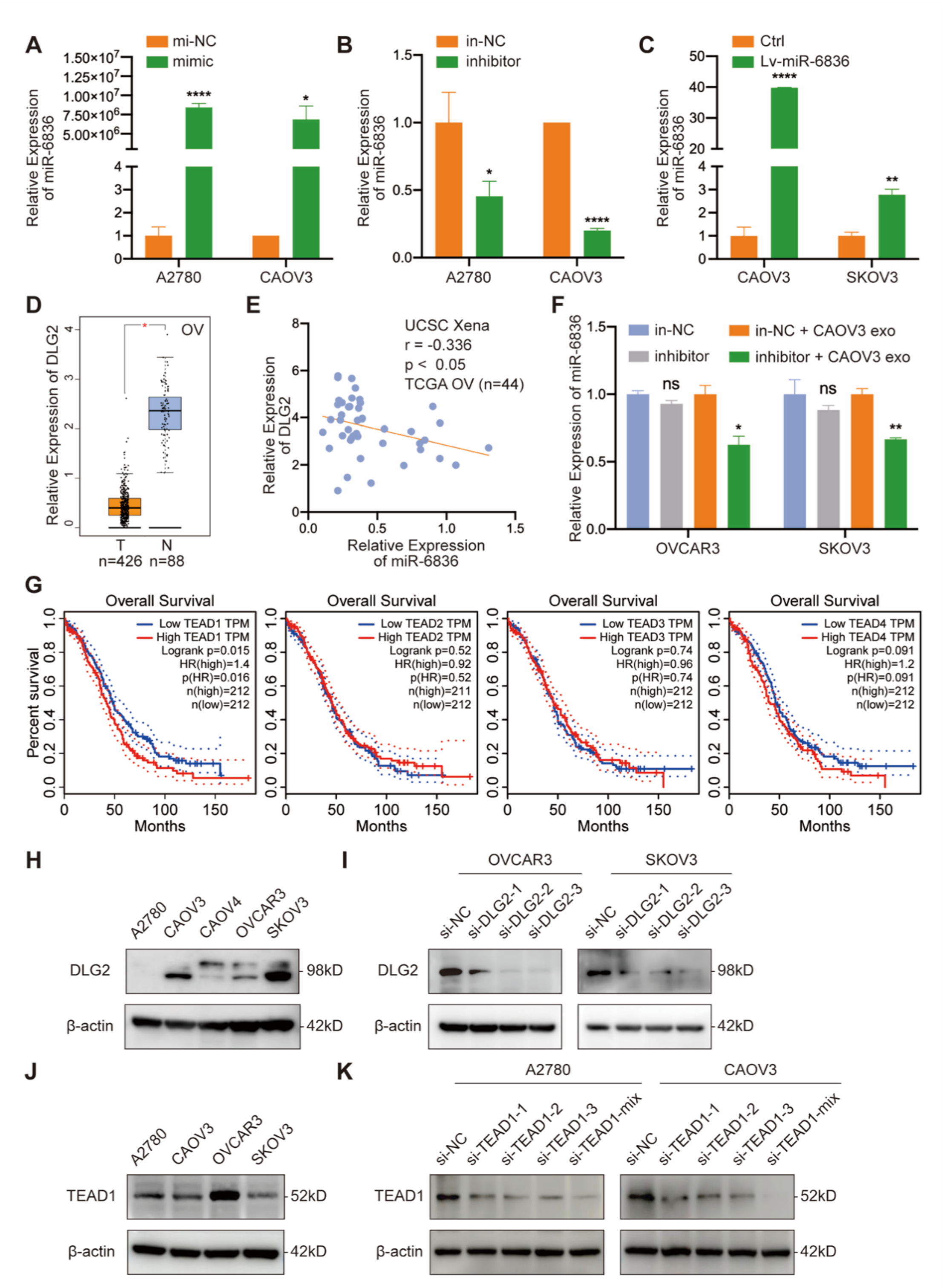

a RT-qPCR analysis of miR-6836 expression in A2780/CAOV3 transfected with mimic/mi-NC.

b RT-qPCR analysis of miR-6836 expression in A2780/CAOV3 transfected with

inhibitor/in-NC.

c RT-qPCR analysis of miR-6836 expression in CAOV3/SKOV3 infected with negative control lentivirus (Ctrl) or lenti-miR-6836 (Lv-miR-6836).

d Relative expression of DLG2 in OV datasets from match TCGA normal and GTEx data showing in box plot exported from GEPIA.

e Spearman correlation analysis between DLG2 mRNA and miR-6836 expression levels in EOC tissues from TCGA (n=44) (UCSC Xena, http://xena.ucsc.edu).

f RT-qPCR analysis of miR-6836 expression in OVCAR3/SKOV3 treated with inhibitor/in-NC and CAOV3 exosomes.

g Kaplan–Meier survival curves analysis of OS in EOC patients with different TEAD family expression level exported from GEPIA.

h Western blot analysis of DLG2 expression in EOC cell lines.

i Western blot analysis of DLG2 expression in OVCAR3/SKOV3 treated with si-NC/si-DLG2.

j Western blot analysis of TEAD1 expression in EOC cell lines.

k Western blot analysis of TEAD1 expression in OVCAR3/SKOV3 treated with si-NC/si-TEAD1.

Results are presented as mean ± SEM; *P<0.05, **P<0.01, ***P<0.001.

Supplementary Figure 2

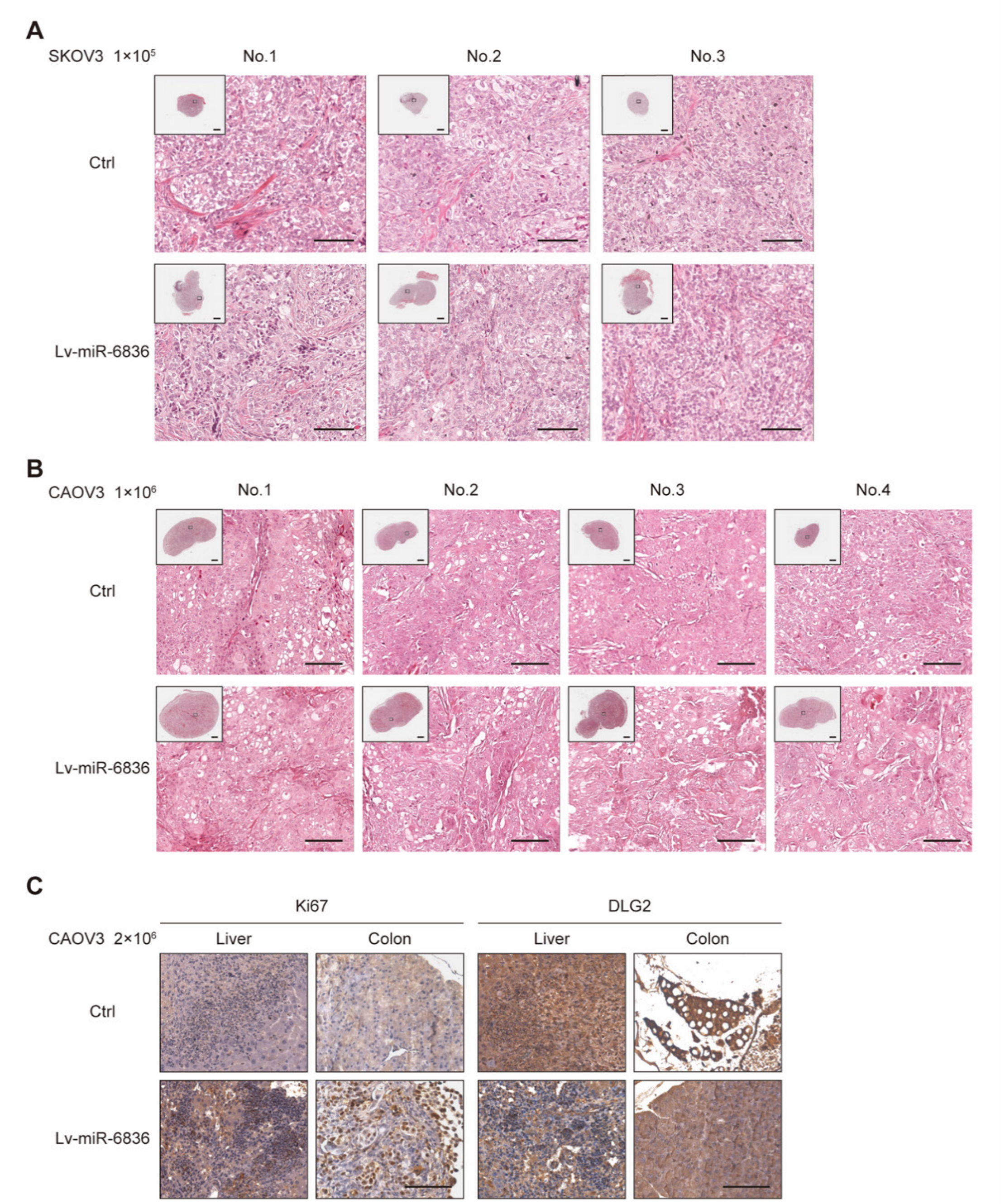

a For spheroid cells tumorigenicity assay, Hematoxylin-eosin (HE) staining of tumor tissues is conducted and representative images of HE staining from Ctrl and Lv-miR-6836 group are shown. Scale bar: upper 1mm; below 100 μm.

b For subcutaneous tumor formation model, HE staining of tumor tissues is conducted and representative images of HE staining from Ctrl and Lv-miR-6836 group are shown. Scale bar: upper 1mm; below 100 μm.

c For peritoneal metastatic model, representative IHC staining of Ki67 and DLG2 in liver and colon metastases. Scale bar, 100 μm.

Abstract Figure

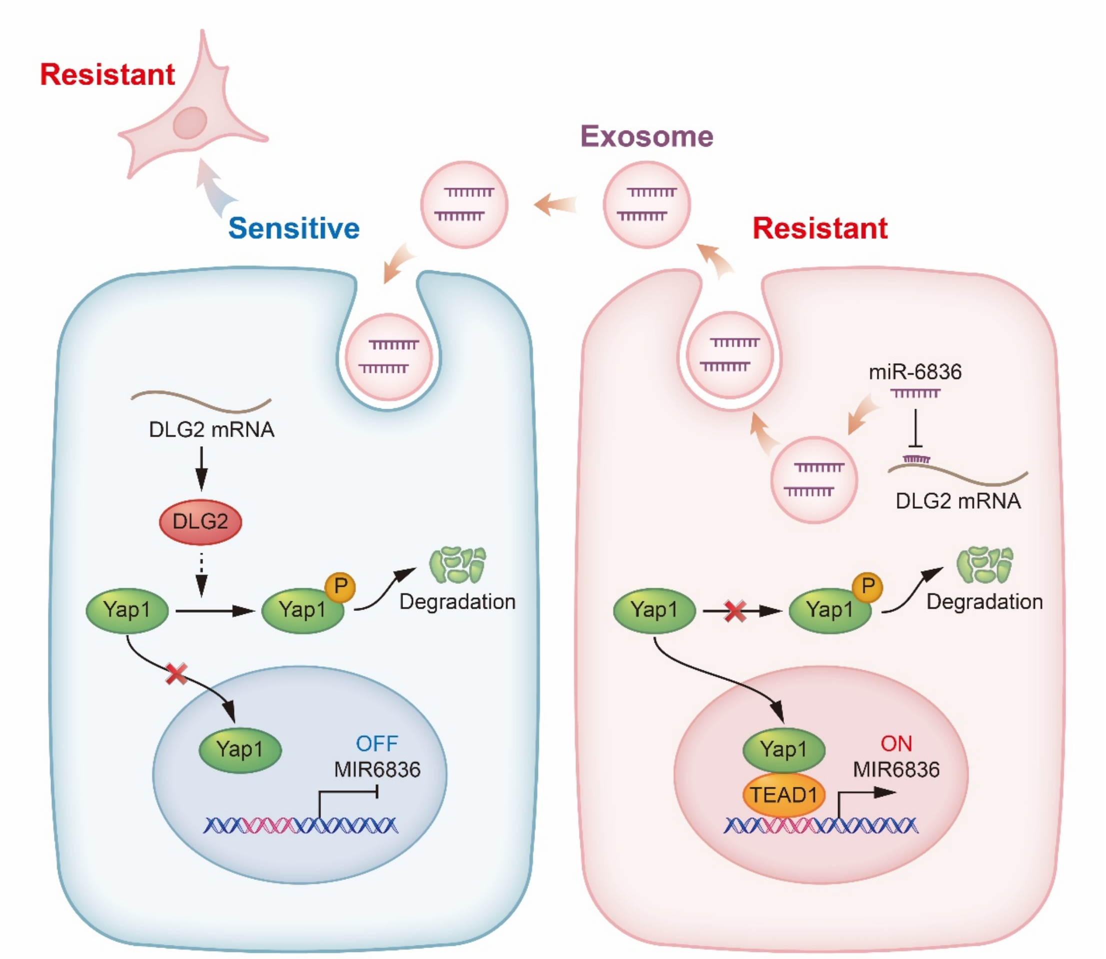

Table S1 Primer sequence

Name	Forward	Reverse
U6	CTCGCTTCGGCAGCACA	AACGCTTCACGAATTTGCGT
External Control	GCTTTGTGGCCATTGTGCAT	CATTTGCCCGTCCCAATGTC
DLG2	GCAGCTTATTCCAAGCTTTGCT	CACGGAGCAAGAAGGGATGT
GAPDH	TGTTGCCATCAATGACCCCTT	CTCCACGACGTACTCAGCG
Primer a	GAAGGGGTATCTTAGGGTCCC	AGGCAGAAGTGGATGGAACG
Primer b	CAGGAGAATGGCGTGAACCC	CTTCGAGCCCAGCAGTCTT
Primer c	GGGTCCTATGTGCGTCCG	GCAGCAGATCCAAGAAGAGGT
